# Older Adults Performed Worse on Cognitive Assessment at Lonelier Moments: Using Ambulatory Assessment Approach

**DOI:** 10.1093/geroni/igab046.3627

**Published:** 2021-12-17

**Authors:** Jee eun Kang, Karra Harrington, Martin Sliwinski

**Affiliations:** The Pennsylvania State University, University Park, Pennsylvania, United States

## Abstract

This study focused on investigating the short-term effect of loneliness on older adults’ cognitive performances in daily life. Loneliness is suggested as a risk factor for cognitive health, but results in previous studies are inconsistent due to the lack of valid measures and limited research design. The attention-depletion hypothesis highlights that acute stress could immediately compromise cognitive ability by consuming attentional resources. Accordingly, this study examined whether loneliness, as one of the stressors related to one’s social relationship, was immediately associated with worse daily cognitive performances in older adults. Using an ecological momentary assessment approach, 311 community-dwelling older adults (Mage=77.5 (range=70-90), 67% female, 45% white) reported their level of loneliness as well as performed cognitive assessment five times a day for 16 days. Multilevel modeling showed that on occasion when participants reported a higher level of loneliness than normal, they performed worse in the processing speed test (p<.01) and the short-term memory binding test (p<.01) during those moments, controlling for age, gender, education, ethnicity, IADL, and retest-practice effect. Moreover, those momentary associations between loneliness and cognitive performances remained significant after controlling for the momentary level of feeling depressed. Unlike the concurrent effect, there was no lagged effect of loneliness on daily cognitive performances. These results suggest that transient but intense feelings of loneliness can function as acute stress and thus, compromise daily cognitive functioning short-term. Results will be discussed in terms of the potential benefit of momentary real-time interventions to lessen feelings of loneliness to maintain older adults’ cognitive functioning.

